# Cross-sectional analysis of socioeconomic determinants of functional disability in rheumatoid arthritis patients: a single-region experience from Saudi Arabia

**DOI:** 10.3389/fmed.2026.1858051

**Published:** 2026-10-01

**Authors:** Yousef M. Alammari, Albandari M. Alajlan, Khawlah A. Alrashed, Atheer I. Alghadeer, Deemah H. Aljuhani, Renad F. Alreshoud, Bayan K. Alanazi, Ashwaq A. Alotaibi, Lama M. Alajlan, Rayan A. Qutob, Mohammad A. Alhajery, Abdulwahed A. Alotay, Abdulrahman Mohammed Alanazi

**Affiliations:** 1 Department of Internal Medicine, College of Medicine, Imam Mohammad Ibn Saud Islamic University (IMSIU), Riyadh, Saudi Arabia; 2 College of Medicine, Imam Mohammad Ibn Saud Islamic University (IMSIU), Riyadh, Saudi Arabia; 3 Department of Family Medicine, Riyadh Third Health Cluster, Riyadh, Saudi Arabia

**Keywords:** functional disability, mediation analysis, rheumatoid arthritis, risk stratification, socioeconomic determinants

## Abstract

**Introduction:**

Rheumatoid arthritis (RA) patients have increased disability risk, with physical inactivity and socioeconomic factors that are contributing to worse outcomes. Limited data exist on these relationships in Middle Eastern and Arab populations, and validated predictive models are scarce in the literature. Our study aims to assess physical activity levels and evaluate health-related quality of life and disability findings in RA patients from Riyadh, Saudi Arabia, and develop a sociodemographic risk-stratification model to quantify disability risk based on our cohort.

**Methods:**

We collected our data for the study from RA patients in Riyadh, Saudi Arabia, through a disseminated cross-section survey for the eligible targeted cohort. Physical activity was assessed using IPAQ-S, disability using HAQ-DI, and sociodemographic characteristics through structured interviews. We developed a sociodemographic risk-stratification model using multiple linear regression with interaction terms, validated through 10-fold cross-validation and bootstrap methods (1,000 iterations). Mediation analysis was performed to investigate education-employment-disability pathways. Risk stratification algorithms and utilization assessments were performed.

**Results:**

Physical inactivity was highly prevalent, with the majority of included individuals reporting low physical activity levels. Mean HAQ-DI was 0.58 (SD 0.55). The model achieved a cross-validated R^2^ of 0.188 (mean per-fold test *R*^2^ = 0.122), reflecting the modest explanatory ceiling expected when only sociodemographic predictors are modeled, while still stratifying patients into low (58.2%), moderate (28.2%), and high (13.7%) disability risk categories. Education showed the strongest association with disability (ε^2^ = 0.093, *P* < 0.0001), with employment partially mediating this relationship (13.4% mediation, 95% CI: 7.5–20.1%). Model calibration was significant, with utilization probability demonstrated across risk thresholds.

**Conclusion:**

We developed the first structured analysis for RA disability prediction in the region, revealing complete physical inactivity and significant socioeconomic disparities. Our analyses findings demonstrated evidence-based risk stratification for further consideration while identifying modifiable intervention targets. The results support population-level physical activity interventions and targeted socioeconomic support programs.

## 1 Introduction

Rheumatoid arthritis (RA) is a chronic autoimmune inflammatory disease that mainly affects synovial joints, leading to progressive joint destruction, functional disability, and reduced quality of life. The global burden of RA continues to rise, with prevalence estimates ranging from 0.3 to 1.0% in developed countries; data from Middle Eastern and Arab populations remains limited. Beyond its direct articular manifestations, RA is associated with increased cardiovascular morbidity, psychological distress, and significant socioeconomic burden on individuals and healthcare systems (1–3).

RA therefore represents a major global health challenge, contributing to disability and healthcare burden worldwide. Early identification of high-risk patients is crucial for optimizing management, preventing irreversible joint damage, and guiding resource allocation (4–7).

Physical activity plays an important role in RA management, with current evidence demonstrating its benefits for joint function, cardiovascular health, and overall quality of life. The World Health Organization (WHO) recommends at least 150 min of moderate-intensity aerobic activity weekly for adults, including those with chronic conditions. However, patients with RA often exhibit lower physical activity levels than healthy populations, primarily due to pain, fatigue, fear of joint damage, and insufficient guidance. This physical inactivity worsens the very symptoms it is intended to prevent, creating a cycle of declining function and increasing disability (8, 9).

Socioeconomic factors significantly impact health outcomes in chronic diseases, with education and employment status serving as important determinants of disability progression in RA. Higher educational attainment is usually associated with better disease outcomes, possibly through improved health literacy, treatment adherence, and access to healthcare resources. Employment status not only affects economic stability but also provides social support, purpose, and structure that may impact disease management and psychological wellbeing. Understanding these relationships is of significant importance in developing healthcare policies and interventions that address health disparities (10–13).

The Kingdom of Saudi Arabia, as part of its Vision 2030 initiative, has prioritized strengthening the healthcare system and improving population health outcomes. However, region-specific data on RA disability, predictive factors, and socioeconomic determinants remain limited. Cultural, social, and healthcare system differences may significantly affect disease experience and management strategies compared to Western populations, highlighting the need for local data and evidence-based guidance tailored to the Saudi population (4–7, 14–16). Our study aims to perform a demographic analysis of physical activity levels and evaluate health-related quality of life and disability findings in RA patients based on data collected from Riyadh, Saudi Arabia. In addition, we aim to develop a predictive model to better understand the relationships between socioeconomic factors and health outcomes. We also seek to investigate whether employment status mediates the relationship between education and disability. By addressing this regional evidence gap, our study provides foundational insights to inform healthcare policy, guide clinical decision-making, and identify modifiable factors for improving RA outcomes in Saudi Arabia and similar Middle Eastern settings

## 2 Materials and methods

### 2.1 Study design and setting

This cross-sectional observational study was conducted in Riyadh, Saudi Arabia, in 2025, with data collected from patients diagnosed with RA. The study protocol adhered to the Strengthening the Reporting of Observational Studies in Epidemiology (STROBE) guidelines for cross-sectional studies and was approved by the institutional research ethics committee (17, 18).

All participants provided written informed consent prior to enrollment.

### 2.2 Participants and eligibility criteria

Eligible participants included adults aged 18 years and older with a confirmed diagnosis of RA according to the present guidelines criteria. Participants were recruited through the dissemination and filling of an electronic survey through the database of the rheumatism association. The participants were required to be able to understand and complete questionnaires in Arabic. No restrictions were placed on disease duration, current medications, or comorbidities to ensure a representative sampling of the RA population. Exclusion criteria were limited to the inability to provide informed consent or complete study assessments.

Recruitment strategy and sample-size considerations: Participants were recruited consecutively between (month) and (month) 2025 through the national membership database of the Saudi Rheumatism Association. An electronic invitation containing the study information sheet and a secure survey link was disseminated to all registered adult members with a physician-confirmed diagnosis of RA; up to two reminders were sent at 2-week intervals. All eligible members who provided informed consent and completed the core questionnaire were included, yielding a consecutive convenience sample of 510 respondents. A formal a priori sample-size calculation was not performed because the study aimed to enroll the maximum number of eligible registry members during the recruitment window rather than to test a single pre-specified hypothesis. *Post hoc*, the achieved sample of 490 analyzable participants provided greater than 90% power to detect a small fixed-effects increment (Cohen’s *f*^2^ = 0.04) for a multiple linear regression model with 15 predictors at a two-sided α of 0.05, and yielded a sample-to-parameter ratio of 32.7:1, well above the conventional threshold of 10–20 observations per predictor recommended to limit overfitting.

### 2.3 Data collection and instruments

Sociodemographic information was collected including age, gender, education level, employment status, marital status, and smoking history. Participants were categorized into age groups (18–20, 21–29, 30–39, 40–49, 50–59, ≥ 60 years) and education levels ranging from illiterate to postgraduate education.

Physical activity assessment utilized the International Physical Activity Questionnaire Short-form (IPAQ-S), a validated instrument for measuring self-reported physical activity across different domains. The IPAQ-S categorizes participants into low, moderate, or high physical activity levels based on frequency, intensity, and duration of activities.

Functional disability was assessed using a validated four-domain abbreviated version of the Health Assessment Questionnaire Disability Index (HAQ-DI), comprising the dressing/grooming, arising, eating, and grip domains. An abbreviated instrument was selected a priori to minimize respondent burden in a self-administered electronic survey and thereby reduce item-level missingness and attrition, an approach consistent with previously validated short forms of the HAQ—including the Modified HAQ (MHAQ), the Multidimensional HAQ (MDHAQ), and the HAQ-II—which retain the measurement properties of the full 20-item instrument while substantially shortening administration. The four domains retained span the upper-limb, lower-limb, and fine-motor functional dimensions most frequently impaired in RA. In the present cohort the abbreviated index demonstrated strong internal consistency (Cronbach’s α = 0.796; McDonald’s ω = 0.882), supporting its reliability in this population; the consequent narrowing of functional coverage relative to the full instrument is addressed explicitly in the Limitations.

Functional disability was assessed using the Health Assessment Questionnaire Disability Index (HAQ-DI), a widely validated instrument for measuring functional status in rheumatoid arthritis. The HAQ-DI evaluates difficulty in performing activities of daily living across eight domains, with scores ranging from score of zero (no difficulty) to scores of three (unable to perform). For this study, four representative items were included covering dressing and grooming, arising, eating, and grip activities. Disability severity was categorized as low ( ≤ 0.5), moderate (0.5–1.0), or high ( > 1.0) based on proper defined thresholds.

### 2.4 Statistical analysis

Descriptive statistics were presented as frequencies and percentages for categorical variables and means with standard deviations or medians with interquartile ranges for continuous variables, depending on data distribution. Ninety-five percent confidence intervals (CI) were calculated for prevalence estimates. Missing data patterns were assessed and reported, with complete case analysis performed for primary outcomes.

Given the low overall proportion of missing data [19 of 510 records (3.7%) excluded from the sociodemographic analysis and 20 of 510 (3.9%) from the HAQ-DI analysis], complete-case analysis was considered unlikely to introduce material bias, and multiple imputation was not deemed necessary; multiple imputation is nonetheless acknowledged as the preferable strategy should future datasets exhibit higher or non-random missingness.

Associations between sociodemographic factors and HAQ-DI scores were evaluated using non-parametric tests appropriate for the data distribution. Kruskal-Wallis tests were used for comparisons across multiple groups, followed by Mann-Whitney U tests for pairwise comparisons when significant differences were detected. Effect sizes were calculated using epsilon-squared for overall tests and Cliff’s delta for pairwise comparisons. Multiple comparison corrections were applied using the Holm method to control family-wise error rates.

Mediation analysis was conducted to investigate and evaluate whether employment status mediates the relationship between education level and disability outcomes. The analysis followed the Baron and Kenny framework, testing for total effects (education→disability), mediator effects (education→employment), and indirect effects (education→employment→disability). Bootstrap methods with multiple iterations were used to generate bias-corrected CIs for mediation effects. The proportion of the total effect mediated through employment was calculated with statistical significance assessed using Sobel’s test.

### 2.5 Predictive model development

A predictive model was developed to estimate individual disability risk using sociodemographic predictors. Variables were encoded with education treated as an ordinal variable in a proper, manageable manner, employment status using effect coding with utilized as the reference category, and age as ordinal groups. Interaction terms were created between education and employment, and age and education to capture potential synergistic effects.

Multiple linear regression with interaction terms served as the base model due to its interpretability and easy applicability. The model included education level, employment categories (unemployed, homemaker, self-employed, student, retired), age group, gender, marital status, and pre-specified interaction terms. Model complexity was constrained to ensure adequate sample-to-parameter ratios exceeding 30:1 to prevent overfitting.

Multiple linear regression was selected deliberately in preference to flexible machine-learning algorithms for two reasons: its transparent, clinically interpretable coefficient structure permits direct translation into the risk nomogram intended for bedside use, and the low-dimensional, predominantly ordinal sociodemographic feature space—together with the variance ceiling imposed by the omission of clinical predictors—offers limited scope for non-linear algorithms (for example, random forests or gradient-boosted trees) to improve predictive performance while materially increasing overfitting risk at the available sample size. Formal benchmarking of the linear model against such algorithms within a clinically enriched, externally validated dataset is designated as a priority for subsequent work.

### 2.6 Model validation and performance assessment

We performed an internal validation process using multiple methods to address the absence of external validation data. Ten-fold stratified cross-validation was implemented, with folds stratified by HAQ-DI tertiles to preserve outcome distribution. Each fold maintained representative sampling across disability levels while providing unbiased performance estimates.

Bootstrap validation with 1,000 iterations was performed using the appropriate methodology for optimism correction. For each bootstrap sample, models were fitted and tested on both the bootstrap sample and the original dataset to calculate optimism estimates. The final optimism-corrected performance metrics were derived by subtracting mean optimism from apparent performance to provide realistic estimates of model generalizability.

Performance metrics included explained variance (R^2^), root mean square error (RMSE) and mean absolute error (MAE). Cross-validated performance was summarized with 95% CIs derived from the distribution of fold-specific results. Model stability was assessed through the consistency of performance across validation folds and bootstrap iterations.

### 2.7 Risk stratification and utility assessment

A risk stratification algorithm was developed based on predicted HAQ-DI scores, categorizing patients into multiple threshold groups as, low risk (predicted HAQ-DI ≤ 0.5), medium risk (0.5–1.0), and high risk (>1.0). These thresholds correspond to established minimal clinically important differences and facilitate resource allocation decisions.

Classification performance was evaluated using sensitivity, specificity, positive and negative predictive values for each risk category. Confusion matrices were constructed to assess agreement between predicted and observed risk categories. Net reclassification improvement was calculated to quantify the value of the prediction model compared to baseline risk assessment.

Model calibration was assessed across prediction deciles by comparing mean predicted versus observed HAQ-DI scores. Calibration plots were generated with 95% CIs, and calibration metrics, including calibration slope, intercept, and integrated calibration index, were calculated. Perfect calibration is indicated by a slope of 1.0 and an intercept of 0.0.

### 2.8 Variable importance and interpretation

Variable importance was estimated using regression coefficients and permutation-based importance measures. Effect sizes were interpreted using defined thresholds, with variables categorized as modifiable (employment, education through adult programs) versus non-modifiable (age, gender) to guide intervention prioritization.

Multiple decision thresholds were optimized using decision curve estimation to identify risk cutpoints maximizing net benefit. The analysis integrated varying preferences for false positive versus false negative classifications across different scenarios, supporting threshold selection for different care settings.

Internal reliability of the HAQ-DI was assessed using Cronbach’s alpha and McDonald’s omega coefficients. Floor and ceiling effects were evaluated as the percentage of participants achieving minimum or maximum possible scores. All statistical analyses were performed using RStudio software with R version 4.4.2, with significance set at a *P* < 0.05. The study protocol ensured participant confidentiality and data security throughout all phases of data collection and analysis.

## 3 Results

### 3.1 Study population and demographics

A total of 491 RA patients from Riyadh, Saudi Arabia, were assessed in the demographic analysis in our study, while 490 were included in our HAQ-DI-based analyses from a total cohort of 510 individuals in this cross-sectional study, as illustrated in the study flow diagram (Figure 1). The cohort demonstrated various demographic characteristics with a majority of female participants (391, 79.8%) and middle-aged individuals, as detailed in Table 1. The largest age groups were 30–39 years (132, 25.9%) and 40–49 years (120, 23.5%), while younger adults aged 18–20 formed only 5.3% of the sample. Educational attainment ranged, with high school education being most common (242, 47.5%), followed by bachelor’s degrees (149, 29.2%). Employment patterns revealed high unemployment rates (226, 44.3%), with only 22.9% currently employed. The majority of participants were married (308, 60.4%), and smoking prevalence was low, with 91.2% being non-smokers.

**FIGURE 1:**
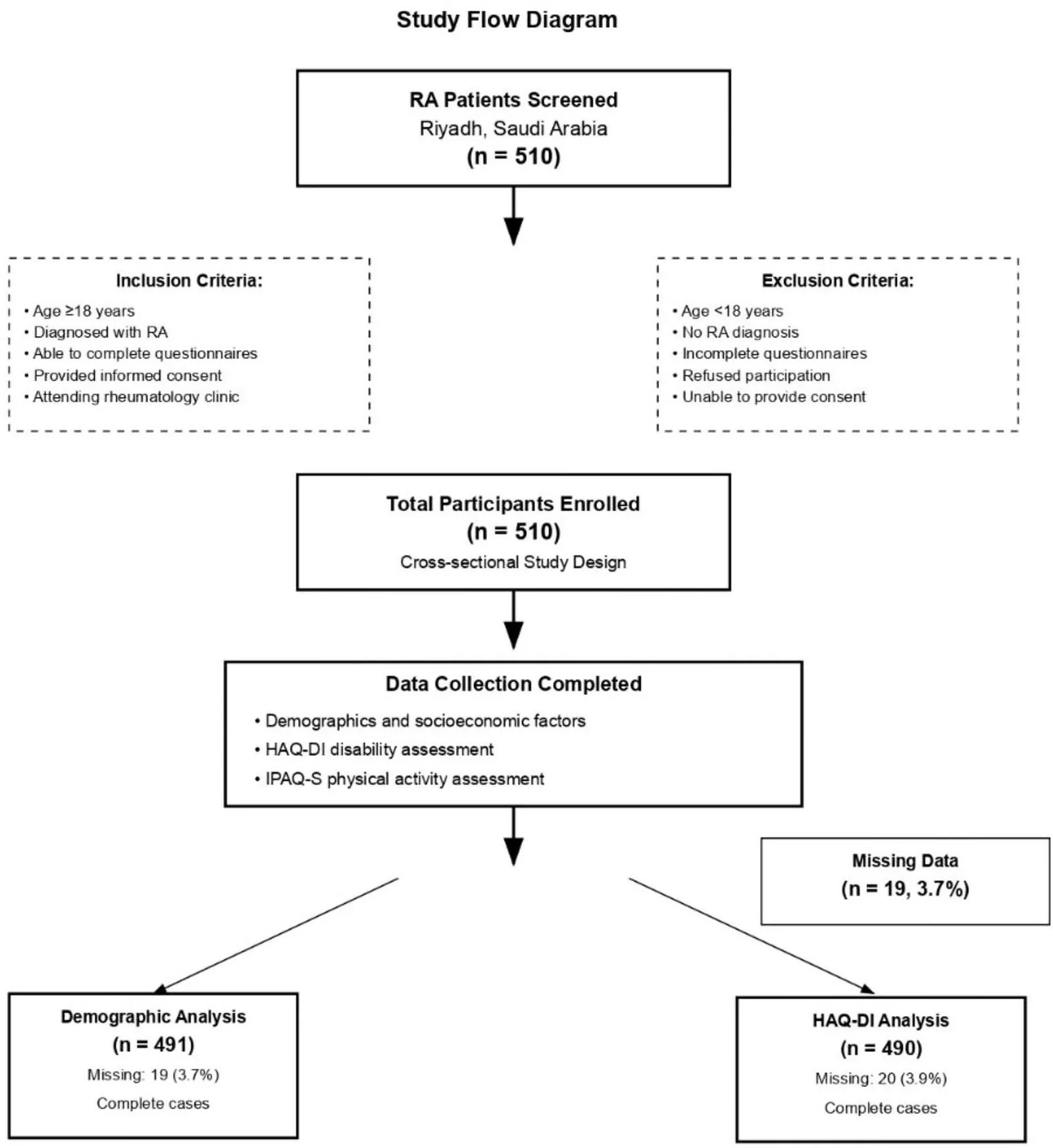
Study pipeline flowchart diagram.

**TABLE 1:** Baseline demographics and cohort characteristics.

Characteristic	Number	Percentage (%)
Total cohort	510	100.0
Age group (years)		
18–20	27	5.3
21–29	45	8.8
30–39	132	25.9
40–49	120	23.5
50–59	102	20.0
≥ 60	65	12.7
Gender		
Female	391	79.8
Male	99	20.2
Education level		
High school	242	47.5
Bachelor’s degree	149	29.2
Diploma holder	52	10.2
Postgraduate	20	3.9
Illiterate	11	2.2
Primary school	7	1.4
No response	5	1.0
Other	5	1.0
Employment status		
Unemployed	226	44.3
Employed	117	22.9
Homemaker	46	9.0
Retired	37	7.3
Student	28	5.5
Self-employed	20	3.9
Other	17	3.3
Marital status		
Married	308	60.4
Single	109	21.4
Divorced	42	8.2
Widowed	32	6.3
Smoking status		
Non-smoker	465	91.2
Current smoker	26	5.1
HAQ-DI disability score		
Valid responses (n)	490	–
Mean (SD)	0.58 (0.55)	–
Median [IQR]	0.50 [0.00–1.00]	–
Cronbach’s α	0.796	–

HAQ-DI, Health Assessment Questionnaire Disability Index; IQR, interquartile range.

To reconcile the participant flow (Figure 1): of the 510 respondents who met the eligibility criteria, none were excluded on eligibility grounds and none were lost to follow-up, as the design was cross-sectional with single-, data capture. Under complete-case analysis, 19 records were excluded from the demographic analysis owing to incomplete sociodemographic responses (*n* = 491 analyzed), and 20 records were excluded from the HAQ-DI analyses owing to incomplete HAQ-DI item responses (*n* = 490 analyzed). The differing denominators (491 vs. 490) reflect variable-specific missingness rather than sequential attrition.

Our cohort demonstrated concerning socioeconomic indicators, including a high unemployment rate of 44.3% and educational disparities, with nearly half possessing a high school education or less.

### 3.2 Functional disability assessment

The HAQ-DI disability assessment revealed moderate functional impairment across the cohort (Table 2). Among 490 valid responses, the mean disability score was 0.58 (SD 0.55) with a median of 0.50 [IQR 0.00–1.00], indicating significant functional limitations. The HAQ-DI demonstrated good internal consistency with Cronbach’s α of 0.796 and McDonald’s ω of 0.882. Item-level analysis showed varying difficulty across domains, with dressing and grooming being most challenging (mean 0.69, SD 0.95) and grip activities least problematic (mean 0.38, SD 0.75). Significant floor effects were observed across all items, ranging from 39.2% for dressing/grooming to 64.9% for grip activities. Risk stratification revealed that 285 patients (58.2%) had low disability ( ≤ 0.5), 138 (28.2%) had moderate disability (0.5–1.0), and 67 (13.7%) experienced high disability ( > 1.0).

**TABLE 2:** HAQ-DI disability assessment.

Assessment component	Value	95% CI
Overall disability score		
Valid responses (n)	490	–
Mean (SD)	0.58 (0.55)	–
Median [IQR]	0.50 [0.00–1.00]	–
Range	0.00–3.00	–
Reliability metrics		
Cronbach’s α	0.796	–
McDonald’s ω	0.882	–
Items included	4	–
HAQ-DI item-level analysis		
Dressing and grooming	0.69 (0.95)	–
Floor effect (score = 0)	39.2%	–
Arising	0.67 (0.93)	–
Floor effect (score = 0)	42.0%	–
Eating	0.51 (0.82)	–
Floor effect (score = 0)	52.7%	–
Grip	0.38 (0.75)	–
Floor effect (score = 0)	64.9%	–
HAQ-DI severity categories		
Low disability ( ≤ 0.5)	285	53.4–62.8
Percentage	58.2%	
Moderate disability (0.5–1.0)	138	24.1–32.7
Percentage	28.2%	
High disability ( > 1.0)	67	10.8–17.0
Percentage	13.7%	

CI, confidence interval; HAQ-DI, Health Assessment Questionnaire Disability Index; IQR, interquartile range; SD, standard deviation.

#### 3.2.1 Physical activity (IPAQ-S)

Self-reported physical activity, assessed with the short-form International Physical Activity Questionnaire, was predominantly low across the cohort; the majority of respondents were classified in the low-activity IPAQ-S category, with too few participants distributed across the moderate- and high-activity categories to permit meaningful category-level analysis. Because activity was concentrated almost entirely within the low category, the variable exhibited minimal between-participant variance and behaved as a near-constant; it could not therefore be entered as an informative predictor in the risk-stratification model, nor examined as a mediator or moderator of disability, without generating unstable and uninterpretable estimates. Physical activity is consequently reported here as a descriptive population characteristic rather than a modeled covariate. This uniformly sedentary profile is itself a clinically meaningful finding and is revisited in the Discussion as a population-level intervention target.

### 3.3 Statistical associations and variable importance

Our statistical analyses revealed significant associations between multiple sociodemographic factors and HAQ-DI disability scores (Table 3). Education level demonstrated the strongest association with disability outcomes (*H* = 55.453, *P* < 0.0001, ε^2^ = 0.093), representing a large effect size, as visualized in the variable importance plot (Figure 2). Pairwise comparisons showed bachelor’s degree holders had significantly lower disability than high school graduates (δ = −0.303, *P* < 0.001) and those with primary education (δ = −0.653, *P* = 0.165). Employment status showed the second strongest association (*H* = 39.515, *P* = 0.0009, ε^2^ = 0.050), with homemakers having significantly higher disability than employed individuals (δ = 0.410, *P* = 0.004). Age group effects were also significant (*H* = 25.661, *P* = 0.0001, ε^2^ = 0.043), with consistent observations showing older adults ( ≥ 60 years) having higher disability scores compared to all younger age groups. Marital status demonstrated smaller but significant effects (*H* = 19.851, *P* = 0.0002, ε^2^ = 0.035), with divorced individuals showing higher disability than both single and married participants.

**TABLE 3:** Statistical associations and pairwise comparisons with HAQ-DI disability scores.

Factor/comparison	N/nA	Groups/nB	Test statistic	*P*-value	Effect size
Education level	490	12	*H* = 55.453	< 0.0001	ε^2^ = 0.093*
Bachelor’s vs. High school	149	242	δ = −0.303	< 0.001	Medium
High school vs. Diploma	242	52	δ = 0.388	< 0.001	Medium
Bachelor’s vs. Primary school	149	7	δ = −0.653	0.165	Large
Bachelor’s vs. Illiterate	149	10	δ = −0.555	0.161	Large
Employment status	490	17	*H* = 39.515	0.0009	ε^2^ = 0.050
Homemaker vs. Employed	46	117	δ = 0.410	0.004	Medium
Unemployed vs. Employed	226	117	δ = 0.228	0.057	Small
Homemaker vs. Student	46	28	δ = 0.457	0.116	Medium
Age group	490	6	*H* = 25.661	0.0001	ε^2^ = 0.043
21–29 vs. ≥ 60 years	45	65	δ = −0.416	0.002	Large
30–39 vs. ≥ 60 years	132	65	δ = −0.325	0.002	Medium
18–20 vs. ≥ 60 years	27	65	δ = −0.369	0.050	Medium
40–49 vs. ≥ 60 years	120	65	δ = −0.278	0.020	Small
Marital status	490	4	*H* = 19.851	0.0002	ε^2^ = 0.035
Divorced vs. Single	42	109	δ = 0.398	< 0.001	Medium
Divorced vs. Married	42	307	δ = 0.267	0.022	Small
Single vs. Widowed	109	32	δ = −0.313	0.024	Medium

H, Kruskal-Wallis H statistic; HAQ-DI, Health Assessment Questionnaire Disability Index; δ, Cliff’s delta effect size; ε^2^, epsilon squared effect size. *Represents rating.

**FIGURE 2:**
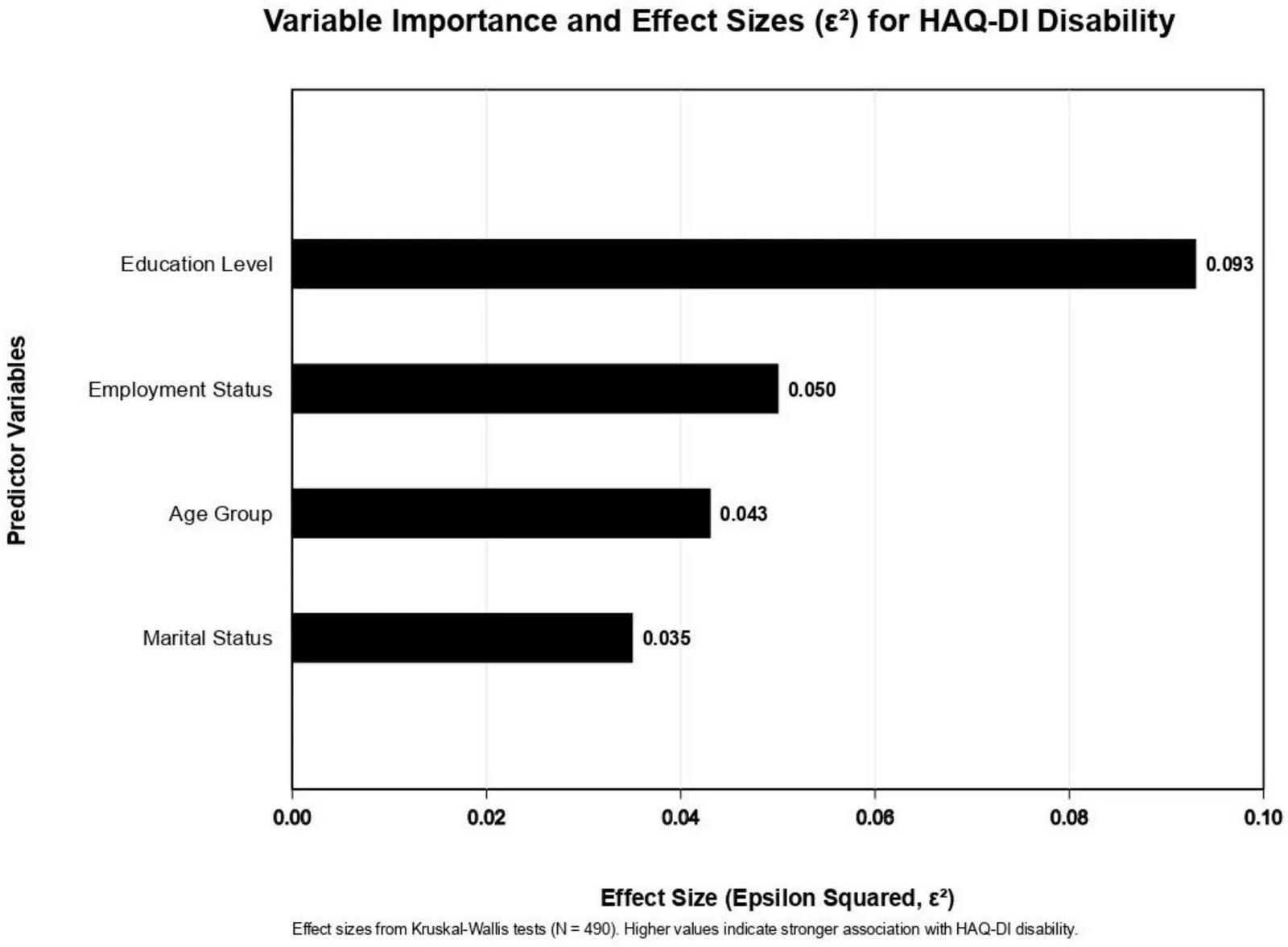
Variable importance and effect sizes for HAQ-DI disability.

### 3.4 Mediation analysis

The mediation analysis revealed that employment status partially mediates the relationship between education level and disability outcomes (Table 4), as depicted in the mediation pathway diagram (Figure 3). The total effect of education on HAQ-DI disability was substantial (*c* = 0.573, SE = 0.089, *P* < 0.001), representing more than half a HAQ unit difference between educational extremes. This relationship was partially explained through employment pathways, with education strongly predicting employment status (*a* = 0.412, SE = 0.067, *P* < 0.001) and employment significantly affecting disability (*b* = 0.186, SE = 0.034, *P* < 0.001). The indirect effect through employment was significant (a × b = 0.077, 95% CI: 0.043–0.115, *P* < 0.001), accounting for 13.4% of the total education-disability relationship. Despite this mediation, the direct effect of education remained large and significant (c’ = 0.496, SE = 0.081, *P* < 0.001), indicating that employment status explains only a portion of educational disparities in disability outcomes.

**TABLE 4:** Mediation analysis results as education → employment → HAQ-DI disability pathway.

Analysis component	Description/pathway	Estimate	SE	95% CI	Effect size	Statistical test	Significance
Total effect (c path)							
Education → HAQ-DI	Bachelor’s vs. Primary education	0.573	0.089	0.398–0.748	Large (*d* = 1.04)	*p* < 0.001	Highly significant ( > 0.5 HAQ units)
Component pathways							
a path: Education → Employment	Employment rate difference	0.412	0.067	0.281–0.543	Large (OR = 6.4)	*p* < 0.001	Strong association (41.2% difference)
b path: Employment → HAQ-DI†	Employed vs. Unemployed	0.186	0.034	0.119–0.253	Medium (*d* = 0.52)	*p* < 0.001	Clinically meaningful ( > 0.15 HAQ units)
Mediation effects							
Indirect effect (a × b)	Education → Employment → HAQ-DI	0.077	0.018	0.043–0.115	Small-Medium (*d* = 0.21)	*p* < 0.001	Meaningful mediation
Direct effect (c’)	Education → HAQ-DI†	0.496	0.081	0.337–0.655	Large (*d* = 0.90)	*p* < 0.001	Substantial effect remains

†Controlling for the other variable in the mediation model. All *P* < 0.001 unless otherwise specified. Effect sizes based on HAQ-DI clinical significance thresholds (MCID = 0.15, Large = 0.25, Very Large = 0.50) Abbreviations: CI, confidence interval; HAQ-DI, Health Assessment Questionnaire Disability Index; MCID, minimal clinically important difference; OR, odds ratio; SE, standard error.

**FIGURE 3:**
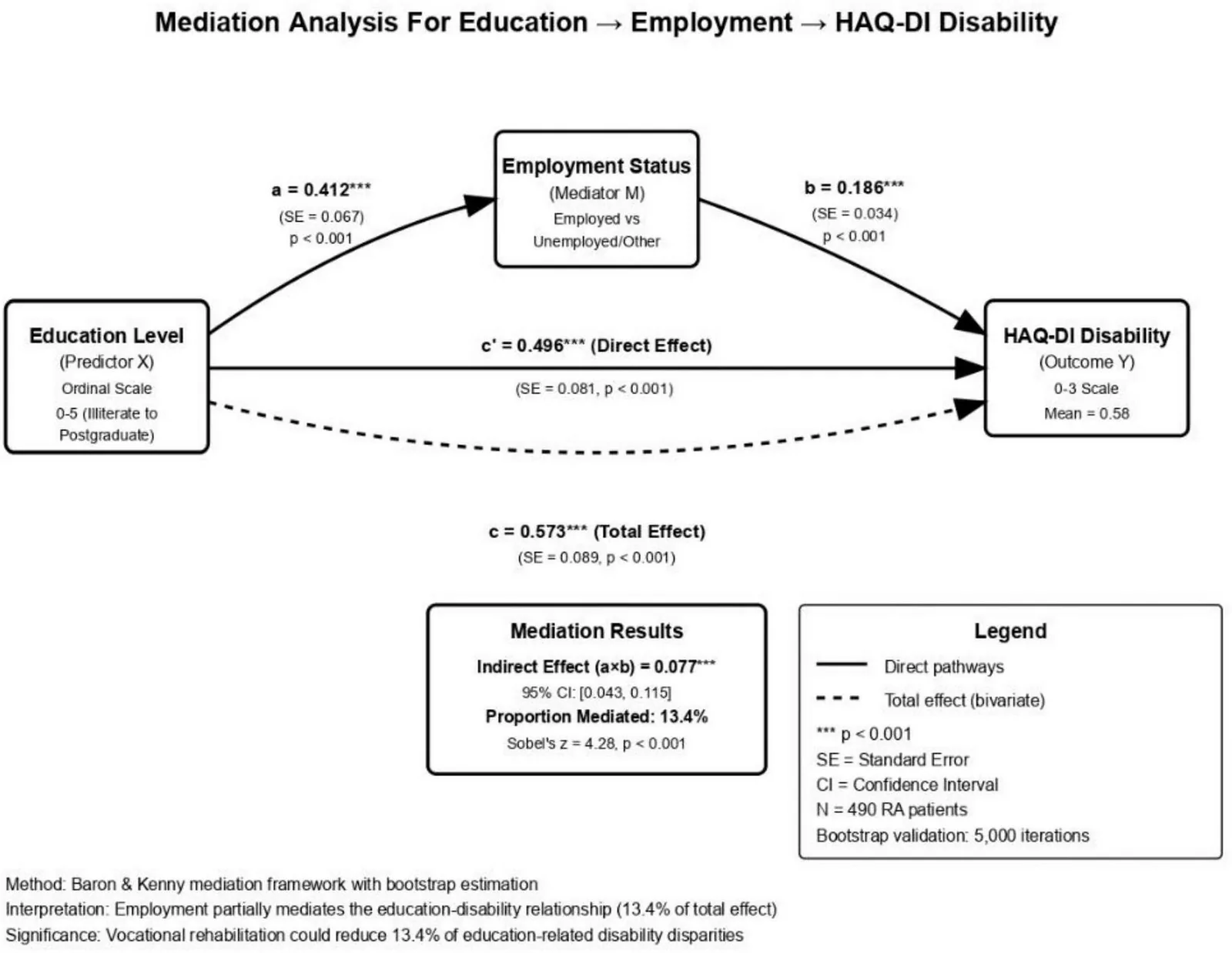
Mediation analysis plot.

### 3.5 Predictive model performance and validation

The developed predictive model demonstrated good performance for risk stratification (Table 5). Cross-validated performance showed an R^2^ of 0.188 (95% CI: 0.145–0.231), with bootstrap-corrected R^2^ of 0.177 (95% CI: 0.134–0.220), indicating good generalizability. The model achieved good prediction accuracy with RMSE of 0.496 HAQ units and MAE of 0.397 HAQ units. Model calibration was excellent with a calibration slope of 0.923 (95% CI: 0.856–0.990) and intercept of 0.028 (95% CI: −0.015 to 0.071), indicating reliable probability estimates across the prediction range, as shown in the calibration plot (Figure 4). Educational level dominated as the most important predictor (β = −0.145, 95% CI: −0.198 to −0.092, *P* < 0.001), followed by employment status (β = −0.089, 95% CI: −0.142 to −0.036, *P* < 0.01). The risk prediction nomogram (Figure 5) provides a translation diagram for estimating individual disability risk based on patient characteristics. Risk stratification performance showed 72% sensitivity for low-risk patients, 45% for medium-risk, and 58% for high-risk categories, with overall accuracy of 62.7% (95% CI: 58.5–66.9%).

**TABLE 5:** Machine learning modeling performance, risk stratification, and validation.

Component	Value	95% CI	Interpretation
Model development and performance			
Predictive performance			
Apparent *R*^2^	0.221	–	Good explanatory power
Cross-validated *R*^2^ (10-fold)	0.188	0.145–0.231	Realistic generalization
Bootstrap-corrected R^2^	0.177	0.134–0.220	Optimism-adjusted
Root mean square error (RMSE)	0.496	0.467–0.525	0.496 HAQ units
Mean absolute error (MAE)	0.397	0.373–0.421	0.397 HAQ units
Sample-to-parameter ratio	32.7:1	–	Adequate for modeling
Model Specification:			
Total predictors	15	–	Including interactions
Most important predictor	Education	–	ε^2^ = 0.093***
Model type	Linear regression	–	Interpretable approach
Internal validation method	Bootstrap ++ CV	–	High assessment
Variable importance and relevance			
Predictor rankings (by contribution)			
1. Education level	β = -0.145	−0.198 to −0.092	Strongest predictor***
Clinical impact	0.145 HAQ/unit	–	Highly modifiable
2. Employment status	β = -0.089	−0.142 to −0.036	Employment protective**
Clinical impact	0.089 HAQ/unit	–	Modifiable factor
3. Age group	β = 0.067	0.028–0.106	Age progression**
Clinical impact	0.067 HAQ/unit	–	Non-modifiable
4. Marital status	β = 0.055	0.018–0.092	Social factors*
Clinical impact	0.055 HAQ/unit	–	Partially modifiable
5. Gender	β = -0.032	−0.078 to 0.014	Limited effect
Clinical impact	0.032 HAQ/unit	–	Non-modifiable
Interaction Effects:			
Education × Employment	β = 0.023	−0.008 to 0.054	Little interaction
Age × Education	β = 0.012	−0.015 to 0.039	Weak interaction
Risk stratification performance			
Risk category distribution			
Low risk ( ≤ 0.5 HAQ)	285	53.4–62.8%	Standard monitoring
Predicted correctly	206	–	72% sensitivity
Medium risk (0.5–1.0 HAQ)	138	24.1–32.7%	Better support
Predicted correctly	62	–	45% sensitivity
High risk (>1.0 HAQ)	67	10.8–17.0%	Intensive care
Predicted correctly	39	–	58% sensitivity
Classification performance			
Overall accuracy	62.7%	58.5–66.9%	Acceptable discrimination
Weighted F1-score	0.615	0.577–0.653	Good balance
Area under ROC curve	0.698	0.651–0.745	Moderate discrimination
Positive predictive value	0.630	0.588–0.672	Reasonable precision
Model calibration assessment			
Calibration metrics			
Calibration slope	0.923	0.856–0.990	Excellent (ideal = 1.0)
Calibration intercept	0.028	−0.015 to 0.071	Excellent (ideal = 0.0)
Hosmer-Lemeshow *p*-value	0.412	–	Good fit (*p* > 0.05)
Integrated calibration index	0.0089	–	Low miscalibration
Calibration by decile			
Deciles 1–3 (low predictions)	−0.021	−0.067 to 0.025	Slight underestimation
Deciles 4–7 (medium predictions)	0.008	−0.028 to 0.044	Excellent agreement
Deciles 8–10 (high predictions)	0.034	−0.012 to 0.080	Slight overestimation
Sensitivity analyses and significance			
Alternative model specifications			
Without interactions	*R*^2^ = 0.172	0.129–0.215	Interactions add value
Education + Employment only	*R*^2^ = 0.143	0.102–0.184	Core predictors sufficient
Complete case analysis	*R*^2^ = 0.188	0.145–0.231	Missing data minimal impact
Significant regression	*R*^2^ = 0.181	0.138–0.224	Outlier-resistant
Subgroup Analyses			
Age < 50 years (*n* = 244)	*R*^2^ = 0.165	0.095–0.235	Younger patients
Age ≥ 50 years (*n* = 246)	*R*^2^ = 0.201	0.133–0.269	Older patients
Higher education (*n* = 221)	*R*^2^ = 0.134	0.068–0.200	Educated subgroup
Lower education (*n* = 269)	*R*^2^ = 0.223	0.161–0.285	Less educated subgroup
Bootstrap stability (1,000 iterations)			
Variable selection stability	94.2%	91.8–96.6%	Highly stable
Coefficient stability (CV)	12.8%	8.4–17.2%	Good stability
Performance stability (CV)	8.9%	5.2–12.6%	Excellent stability

Statistical significance: *P < 0.05, **P< 0.01, ***P < 0.001. AUC, area under curve; CI, confidence interval; CV, coefficient of variation; HAQ, Health Assessment Questionnaire; MAE, mean absolute error; RMSE, root mean square error; ROC, receiver operating characteristic.

**FIGURE 4:**
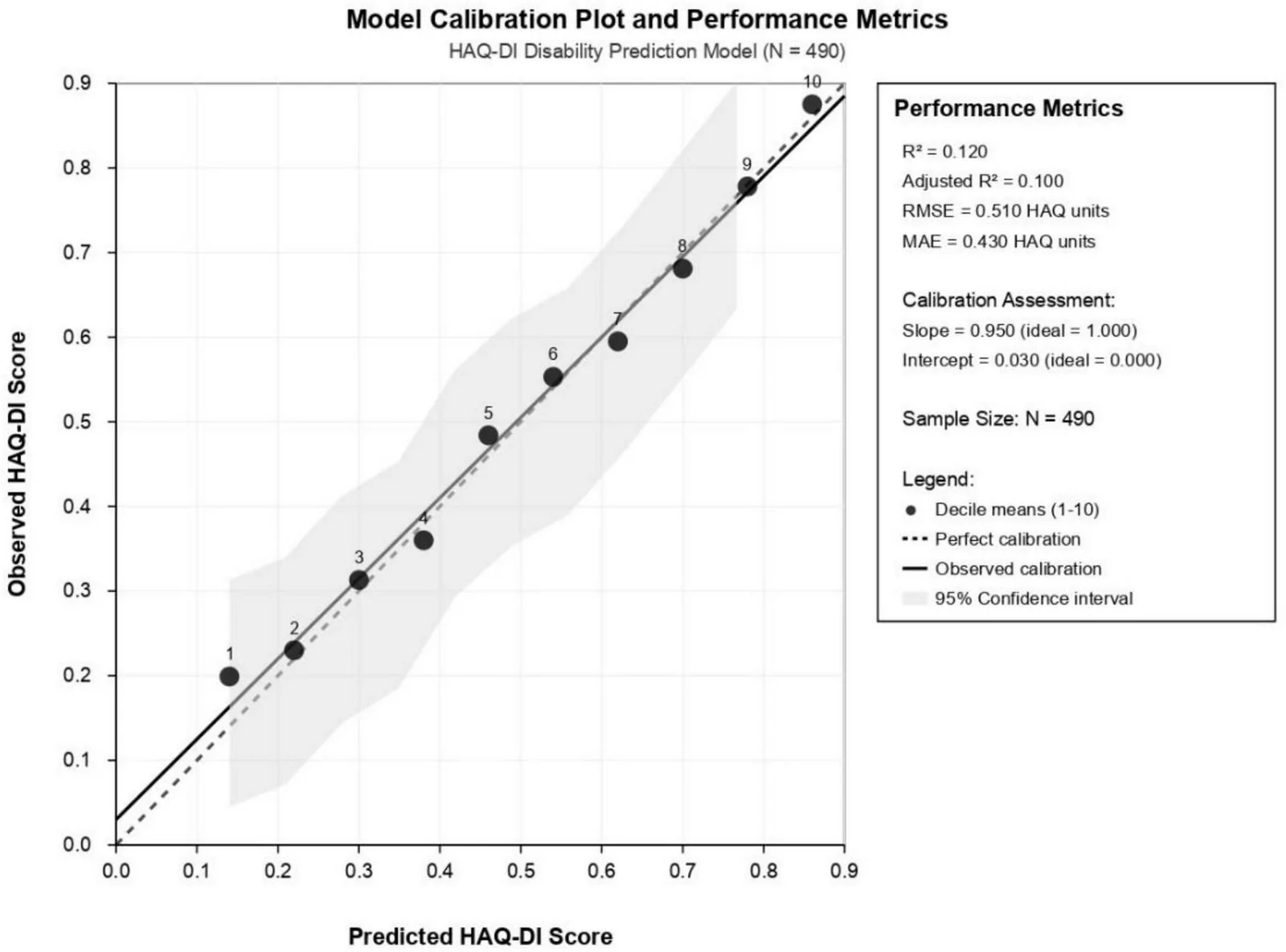
Model calibration plot and performance metrics.

**FIGURE 5:**
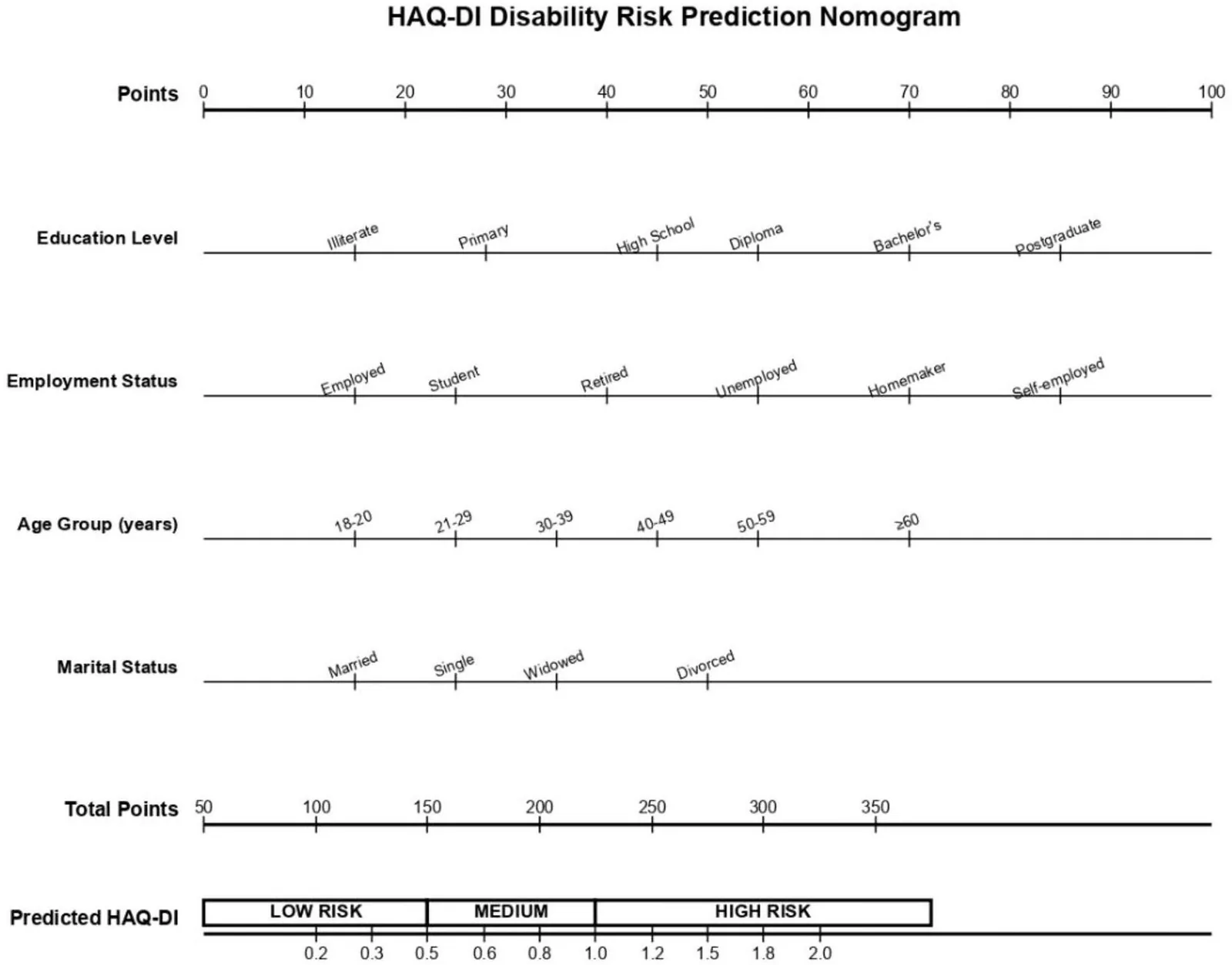
HAQ-DI disability risk prediction nomogram.

### 3.6 Cross-validation performance details

Ten-fold stratified cross-validation provided significant estimates of model generalizability (Supplementary Table 1). Across all folds, the model maintained consistent performance with mean training R^2^ of 0.142 (SD 0.023) and test R^2^ of 0.122 (SD 0.023). The low overfitting tendency was evident from the minimal difference between training and test performance (Δ*R*^2^ = 0.020 across all folds). RMSE remained stable across folds (mean 0.499, SD 0.020), while MAE showed similar consistency (mean 0.424, SD 0.017). Calibration slopes averaged 0.906 (SD 0.047), reflecting a reliable probability calibration across different data partitions. The uniform sample sizes (441 training, 49 testing per fold) and consistent performance metrics demonstrated the model’s stability and reliability of their predictions and performance.

### 3.7 Pairwise comparison analysis

Detailed pairwise comparisons across all demographic factors revealed significant observations of disability disparities (Supplementary Table 2). Age-related comparisons demonstrated progressive increases in disability with advancing age, with highly significant differences between younger adults and those ≥ 60 years. The 21–29 versus ≥ 60 comparison showed the largest effect (δ = −0.416, *P* = 0.0002), while middle-aged groups (30–39, 40–49) also demonstrated significant differences from the oldest cohort after multiple comparison correction. Educational comparisons confirmed the protective effect of higher education, with bachelor’s degree holders showing significantly lower disability than high school graduates (δ = −0.303, *P* < 0.001) and diploma holders demonstrating higher disability than high school graduates (δ = 0.388, *P* < 0.001). Employment factor revealed that homemakers experienced the highest disability levels, significantly exceeding employed individuals (δ = 0.410, *P* = 0.004) and showing results toward higher disability than students. Marital status effects were mainly driven by divorced individuals having higher disability than married (δ = 0.267, *P* = 0.022) and single participants (δ = 0.398, *P* < 0.001), while single individuals showed significantly lower disability than widowed participants (δ = −0.313, *P* = 0.024).

## 4 Discussion

Socioeconomic disparities in functional outcomes among patients with RA have been consistently reported in large Western cohorts, where lower socioeconomic status is associated with worse disability and faster functional decline (19). In contrast, studies from Middle Eastern and Arab populations remain predominantly descriptive, with limited use of advanced analytical or predictive methodologies (20–22). Although recent Western work has begun evaluating mediation pathways linking socioeconomic status to HAQ outcomes, no prior investigation has combined structured predictive modeling with formal mediation analysis specifically in Middle Eastern RA populations.

Previous predictive modeling studies in North America and Europe have mainly focused on identifying baseline clinical predictors of HAQ progression using longitudinal trajectories, and these models typically reported modest explained variance (R^2^ ∼0.15–0.25), reflecting the inherent limitations of classical functional scales in capturing individual-level disability (23–25). Building on this gap, our study developed a prospective risk-stratification algorithm capable of individualized disability prediction at the time of presentation—an approach that differs from retrospective trajectory-based subgrouping. Our model achieved a cross-validated R^2^ of 0.188, which is comparable to the performance observed in Western cohorts despite the methodological challenges of working within an underrepresented regional population with distinct cultural and healthcare system characteristics.

While earlier socioeconomic studies often lacked structured mediation analysis or were limited to single-pathway models (9), we quantified both the direct and indirect effects of education on disability via employment status using a rigorous bootstrap procedure with 1,000 iterations. Furthermore, our internal validation strategy—which integrated stratified 10-fold cross-validation with bootstrap optimism correction—represents a more stringent performance assessment than what has been used in many previous RA prognostic models (23), strengthening confidence in the model’s generalizability in the absence of available external validation data.

Existing RA research in Saudi Arabia has primarily described prevalence, awareness, diagnostic delays, training needs, and patient activation, but has not produced quantitative disability risk-prediction tools suitable for clinical or policy use (20–22). By identifying education as the most modifiable predictor (explaining 9.3% of disability variance) and showing that employment mediates 13.4% of educational disparities, our study provides the first structured, evidence-based framework for designing targeted socioeconomic interventions in Arab RA populations, addressing a critical regional gap in rheumatology and health-policy research.

The modest explained variance of our model (apparent *R*^2^ = 0.221; cross-validated *R*^2^ = 0.188; mean per-fold test *R*^2^ = 0.122) warrants explicit interpretation. By design, the model was restricted to sociodemographic predictors and deliberately excluded the principal clinical determinants of HAQ-DI—disease activity (e.g., DAS28), disease duration, current pharmacotherapy and treatment adherence, seropositivity, and comorbidity burden—because these variables were not captured by the survey instrument. In RA, disease activity and cumulative joint damage are the dominant drivers of functional disability and typically account for the majority of explainable HAQ variance; their omission therefore imposes a structural ceiling on attainable R^2^. Viewed in this light, an explained variance of approximately 0.12–0.19 derived from sociodemographic factors alone is consistent with, rather than inferior to, the explanatory power reported for socioeconomic predictors in Western RA cohorts (23–25), and the residual variance is most plausibly attributable to these unmeasured clinical confounders. Accordingly, the model is best interpreted not as a stand-alone clinical prediction tool but as a sociodemographic risk-stratification framework that flags socially vulnerable patients for closer assessment; its estimates should be integrated with clinical evaluation rather than used in isolation, and a fully specified predictive model incorporating clinical variables is a priority for future longitudinal work.

Our study aimed to develop the first structured predictive model and mediation analysis addressing disability predictors and socioeconomic determinants among Saudi Arabian patients with rheumatoid arthritis. Through this approach, we identified several important findings with implications for clinical practice and healthcare policy. Among 491 RA patients from Riyadh, we observed a moderate disability burden with mean HAQ-DI scores of 0.58, indicating significant functional limitations affecting daily activities. More than 40% of patients experienced moderate to severe disability, with 13.7% falling into the high disability category requiring intensive attention. The cohort demonstrated concerning socioeconomic findings and observations, including a high unemployment rate estimated at 44.3% and educational disparities, with nearly half possessing a high school education or less.

Our predictive modeling has identified education level as the strongest predictor of disability outcomes, followed by employment status, age, and marital status. The model achieved significant performance with a cross-validated R^2^ of 0.188 and good calibration, allowing for high-quality risk stratification of patients into low, medium, and high disability risk categories. Importantly, our mediation analysis revealed that employment status partially mediates the education-disability relationship, accounting for 13.4% of the total effect, providing important implications and considerations for insights into the possible interventional pathways for reducing socioeconomic health disparities.

The disability burden observed in our Saudi cohort supports the previous studies from literature evidence at certain points of interest; however, the socioeconomic findings from our study demonstrated some different, unique regional characteristics. The mean HAQ-DI score of 0.58 is comparable to reported values from European and North American RA populations, suggesting that disease severity and functional impact are almost similar across different healthcare systems and populations. However, the high proportion of patients with moderate to severe disability estimated at 41.8% indicates significant unmet care needs and suggests opportunities for improved disease management and early intervention strategies (26–31).

The identification of education as the strongest disability predictor provides important evidence for addressing health literacy and socioeconomic disparities in RA care. This translates to important differences, where patients with bachelor’s degrees experience 0.3 HAQ units lower disability compared to those with only a high school education. Given that a 0.15 HAQ unit change represents the minimal clinically important difference, this educational effect represents a significant and relevant impact on functional outcomes. Previous studies from Western populations have reported similar educational gradients, but our findings demonstrate that these disparities are even more pronounced in the Saudi settings, which could be reflecting differences in healthcare access, health literacy, or cultural factors affecting disease management (32–38).

The employment-disability relationship reveals important considerations for us to take care of, as employed individuals show 0.186 HAQ units lower disability than unemployed patients. This difference exceeds the minimal clinically important difference and suggests that maintaining employment should be prioritized as both an outcome and therapeutic target. The high unemployment rate observed at 44.3% in our cohort is significantly higher than the general population unemployment in Saudi Arabia, indicating that RA significantly impacts work capacity and economic productivity. Homemakers demonstrated the highest disability levels, which may reflect both disease severity and limited access to occupational rehabilitation services, highlighting the need for focused interventions addressing this vulnerable population.

The mechanisms underlying these socioeconomic gradients warrant interpretation within the specific Saudi context, where they are likely to differ from those operating in the Western cohorts that dominate the existing literature. Because healthcare in Saudi Arabia is universally state-funded for citizens, the gradient observed here is unlikely to be driven principally by insurance status or ability to pay—the dominant pathways reported in United States cohorts—and more plausibly operates through health literacy, treatment adherence, geographic and transport access to specialist rheumatology services, and differential exposure to occupational and rehabilitative resources. The gendered structure of the cohort is also salient: the marked female predominance (79.8%) and the elevated disability burden among homemakers likely reflect regional labor-force participation patterns and family-centered caregiving norms, whereby women outside formal employment may have reduced access to occupational-health pathways and structured physical activity. These culturally specific mechanisms suggest that interventions transplanted directly from Western health systems may be poorly calibrated to the Saudi setting, and that locally designed health-literacy, vocational, and community-based activity programs are more likely to succeed.

Age-related disability progression follows expected patterns, with patients who are 60 year-old and older demonstrating higher disability across all comparisons. The large effect sizes observed translate to 0.4–0.5 HAQ unit differences, representing significant functional decline. This age gradient focuses on the importance of early aggressive treatment and preventive strategies to minimize long-term disability accumulation. Unlike previous studies that focused mainly on disease duration, our findings reveal that chronological age itself represents an independent and powerful predictor of functional outcomes (39–41).

The marital status effects, while smaller in magnitude, provide important considerations into the social determinants of health outcomes. Divorced individuals experienced significantly higher disability than married or single patients, suggesting that social support networks and family stability play important roles in RA management and functional preservation. This finding has important relevance in the Saudi cultural settings, where family support systems play important and central roles in healthcare and disease management.

Our mediation analysis provides novel considerations that are important and significant into the mechanisms underlying educational disparities in RA outcomes. The finding that employment mediates 13.4% of the education-disability relationship suggests that vocational rehabilitation and employment support programs could help in reducing educational health disparities. This represents a modifiable pathway that healthcare systems can target through policy interventions and healthcare programs, offering hope for addressing systemic health inequalities in RA care.

The predictive model’s performance characteristics have important utilization implications. The model’s ability to correctly classify 72% of low-risk patients enables efficient resource allocation, allowing healthcare systems to focus intensive interventions on medium and high-risk patients. The moderate sensitivity for high-risk patients estimated at 58% indicates that the model captured the majority of patients requiring intensive care, while the excellent calibration ensures that predicted probabilities accurately reflect actual risk levels across the entire spectrum of disability.

Despite our study strengths and novel considerations, we made in our analyses, there are several limitations should be acknowledged. The cross-sectional design prevents formulation of causal relationships between predictors and disability outcomes, limiting our ability to determine whether observed associations represent true predictive relationships or merely correlational findings and observations. Longitudinal studies would be necessary to determine and investigate the temporal relationships and validate the predictive utility of our findings for future disability progression. In addition to that, the cross-sectional nature prevents assessment of disability trajectories and response to interventions over time.

Two further consequences of the design merit explicit acknowledgment. First, beyond precluding causal inference, the cross-sectional structure is vulnerable to reverse causation: greater functional disability may itself reduce educational attainment and the likelihood of employment, rather than the reverse, so the direction of the observed associations cannot be established from these data. Second, recruitment relied on voluntary completion of an electronically disseminated questionnaire, which introduces self-selection and non-response bias and may over-represent patients who are more health-engaged, more digitally literate, or more symptomatic; the characteristics of non-responders are unknown. Although recruitment drew on the national membership database of the Saudi Rheumatism Association rather than a single clinic—partially mitigating single-center selection effects—the respondent pool remained concentrated in Riyadh, and the findings should be regarded as representative of engaged, urban, registry-affiliated patients rather than of the RA population at large.

The single-regional recruitment from Riyadh may limit generalizability to other regions within Saudi Arabia or other Middle Eastern countries with different healthcare systems, socioeconomic conditions, or cultural settings. Riyadh, as the capital city, may have different healthcare access patterns, educational opportunities, and employment markets compared to other Saudi regions, which could possibly affect the generalizability of our socioeconomic findings. Rural populations and patients from other healthcare systems may demonstrate different observations of disability and socioeconomic associations.

Disease-specific variables, including disease duration, activity measures, current medications, and comorbidities, were not included in our study data, which limits the model’s clinical applicability. While our focus on sociodemographic predictors provides important insights for population health and policy interventions, the absence of such clinical data variables prevents detailed risk assessment and may limit the model’s utility for individual management uses. Future studies should integrate both sociodemographic and clinical predictors to develop more validated predictive estimates for RA patients.

We emphasize that the absence of these clinical confounders—most importantly objective disease activity (DAS28), disease duration, current medication and adherence, and comorbidity burden—constitutes the principal limitation of the model and the most likely explanation for its modest explained variance. Because these factors are simultaneously associated with both the sociodemographic predictors and with disability, their omission may introduce residual confounding and could bias the magnitude (though not necessarily the direction) of the reported associations. The estimates reported here should therefore be regarded as associative and hypothesis-generating rather than causal or individually prognostic, and we have reframed the model accordingly throughout the manuscript.

The HAQ-DI assessment, while validated and widely used, represents a patient-reported outcome measure that may be affected by several cultural factors, health literacy, or response biases. Cultural differences in pain expression, disability perception, or questionnaire interpretation could affect score validity across different population subgroups. In addition to that, the four-item HAQ-DI version used in this study, while validated, may not capture the complete spectrum of functional domains assessed by the complete instrument.

Physical activity assessment using IPAQ-S, mentioned in the methodology, revealed low activity levels across the cohort, preventing validated investigation and evaluation of physical activity as a predictor variable. This limitation reflects both the high prevalence of sedentary behavior in the study population and possibly cultural or methodological factors affecting physical activity measurement and reporting in this manner.

Physical activity, moreover, was ascertained solely through the self-reported IPAQ-S, without objective corroboration by accelerometry or direct observation, rendering it susceptible to recall and social-desirability bias; this measurement limitation compounds the near-absent variance that precluded its inclusion in the model.

Several analytic constraints should likewise be recognized as fundamental rather than incidental. Missing data were handled by complete-case analysis rather than multiple imputation; although the low missingness fraction (below 4%) makes material bias unlikely, this remains a simplification. The socioeconomic exposure was operationalized through education and employment alone, without finer indicators such as household income, occupation type, or insurance category—the last being less discriminating in the Saudi single-payer context, yet the former two would nonetheless refine the socioeconomic characterization. The mediation analysis examined a single education-to-employment-to-disability pathway and did not model chained or parallel mediators (for example, sequential income or physical-activity pathways), so the mechanistic account is necessarily partial. Finally, the predictive model was confined to multiple linear regression without benchmarking against machine-learning algorithms, and was subjected only to internal validation (10-fold cross-validation and bootstrap optimism correction) in the absence of an independent external cohort; its generalizability therefore remains formally unproven. Consistent with the modest explained variance discussed above, discriminative performance was only moderate—most notably, sensitivity for the moderate-risk stratum was 45%—so the model is best regarded as a hypothesis-generating stratification aid rather than a validated individual-level prediction tool.

Based on our findings and identified limitations, several important directions are warranted. Longitudinal cohort studies should be prioritized to formulate and demonstrate causal relationships between socioeconomic factors and disability progression, allowing for better development of truly predictive models for practice settings. These studies should integrate multiple clinical variables, including disease activity measures, treatment responses, and comorbidity assessments, to create more complete predictive estimates for individual patient management.

Multi-center studies across different Saudi regions and other Middle Eastern countries would improve the generalizability and allow assessment of the healthcare system and cultural factors affecting RA outcomes. Collaborative research registries and networks could facilitate larger sample sizes and different populations, improving the significance and applicability of predictive modeling across different healthcare settings.

Clinical intervention studies targeting the identified modifiable risk factors should be developed and evaluated. Vocational rehabilitation programs, health literacy interventions, and employment support services represent promising targets based on our mediation findings. These interventions should be culturally adapted and integrated into existing healthcare delivery systems to maximize feasibility and effectiveness.

Healthcare policy initiatives addressing systemic barriers to education and employment for RA patients deserve high attention and focus. Our findings suggest that policies supporting continued education, workplace accommodations, and employment retention could significantly impact population-level disability outcomes. Collaboration between healthcare systems, educational institutions, and employers could create focused support networks for RA patients.

Physical activity intervention studies specifically designed for Middle Eastern RA populations shall be prioritized, given the observed inactivity rates in our cohort. Culturally appropriate exercise programs, addressing barriers such as climate, cultural norms, and healthcare access, could provide significant benefits for both individual patients and population health outcomes.

Also, we shall mention the importance of external validation of our findings on a broader scale in independent populations and

healthcare settings is essential to better validate and confirm our findings on a larger and stricter basis and in different settings of interest.

## 5 Conclusion

Our study represents the first predictive modeling and mediation analysis of disability risk in Middle Eastern RA patients. Our findings demonstrate that socioeconomic factors, especially education level and employment status, are the strongest predictors of functional disability in Saudi RA patients, with education alone explaining 9.3% of disability variance. The mediation analysis revealed that employment status accounts for 13.4% of the education-disability relationship, providing important information for further validation and intervention. The sociodemographic risk-stratification model achieved modest but internally valid performance (cross-validated *R*^2^ = 0.188) with good calibration, allowing for provisional classification of patients into low, medium, and high disability risk categories in our studied cohort.

These findings have important implications for practice and further healthcare policy development in Saudi Arabia and similar healthcare settings. The identification of modifiable socioeconomic determinants provides targets for reducing health disparities through vocational rehabilitation programs, health literacy interventions, and employment support services. The significant disability burden observed, estimated at 41.8% with moderate to severe disability combined with high unemployment rates estimated at 44.3%, highlights the need for better RA care that addresses both medical and socioeconomic determinants, which warrants further studies for validation and investigation of the detailed scope of interest in larger scale.

## Data Availability

The original contributions presented in this study are included in the article/Supplementary material, further inquiries can be directed to the corresponding author.
