# Supplementary material for: Cross-sectional analysis of socioeconomic determinants of functional disability in rheumatoid arthritis patients: a single-region experience from Saudi Arabia
Source: Front Med (Lausanne). 2026 Oct 1;13:1858051. doi: 10.3389/fmed.2026.1858051 (PMC13668530; doi:10.3389/fmed.2026.1858051)
Supplement: Supplementary file 2 [file Table_2.docx]

**Supplementary Table 2:** Complete Pairwise Comparison Matrix.

| **Factor** | **Comparison** | **nA** | **nB** | **Cliff's δ** | **p (Unadj)** | **p (Holm)** | **Interpretation** |
| --- | --- | --- | --- | --- | --- | --- | --- |
| **AGE GROUP** | 18-20 vs 21-29 | 27 | 45 | 0.036 | 0.797 | 1.000 | No difference |
|  | 18-20 vs 30-39 | 27 | 132 | -0.017 | 0.886 | 1.000 | No difference |
|  | 18-20 vs 40-49 | 27 | 120 | -0.086 | 0.476 | 1.000 | No difference |
|  | 18-20 vs 50-59 | 27 | 101 | -0.248 | 0.046 | 0.370 | Trend toward difference |
|  | **18-20 vs ≥60** | **27** | **65** | **-0.369** | **0.005** | **0.050** | **Significant difference*** |
|  | 21-29 vs 30-39 | 45 | 132 | -0.060 | 0.536 | 1.000 | No difference |
|  | 21-29 vs 40-49 | 45 | 120 | -0.134 | 0.175 | 1.000 | No difference |
|  | **21-29 vs 50-59** | **45** | **101** | **-0.298** | **0.004** | **0.044** | **Significant difference*** |
|  | **21-29 vs ≥60** | **45** | **65** | **-0.416** | **0.0002** | **0.002** | **Highly significant**** |
|  | 30-39 vs 40-49 | 132 | 120 | -0.058 | 0.416 | 1.000 | No difference |
|  | **30-39 vs 50-59** | **132** | **101** | **-0.216** | **0.004** | **0.045** | **Significant difference*** |
|  | **30-39 vs ≥60** | **132** | **65** | **-0.325** | **0.0002** | **0.002** | **Highly significant**** |
|  | 40-49 vs 50-59 | 120 | 101 | -0.170 | 0.027 | 0.244 | Trend toward difference |
|  | **40-49 vs ≥60** | **120** | **65** | **-0.278** | **0.002** | **0.020** | **Significant difference*** |
|  | 50-59 vs ≥60 | 101 | 65 | -0.101 | 0.270 | 1.000 | No difference |
| **EDUCATION LEVEL** | **Bachelor's vs High school** | **149** | **242** | **-0.303** | **<0.001** | **<0.001** | **Highly significant**** |
|  | Bachelor's vs Diploma | 149 | 52 | 0.106 | 0.237 | 1.000 | No difference |
|  | Bachelor's vs Postgraduate | 149 | 20 | 0.030 | 0.824 | 1.000 | No difference |
|  | Bachelor's vs Illiterate | 149 | 10 | -0.555 | 0.003 | 0.161 | Trend (not significant) |
|  | Bachelor's vs Primary | 149 | 7 | -0.653 | 0.003 | 0.165 | Trend (not significant) |
|  | **High school vs Diploma** | **242** | **52** | **0.388** | **<0.001** | **<0.001** | **Highly significant**** |
|  | High school vs Postgraduate | 242 | 20 | 0.314 | 0.018 | 1.000 | Not significant after correction |
|  | High school vs Illiterate | 242 | 10 | -0.259 | 0.161 | 1.000 | No difference |
|  | High school vs Primary | 242 | 7 | -0.445 | 0.043 | 1.000 | Not significant after correction |
|  | Diploma vs Postgraduate | 52 | 20 | 0.065 | 0.655 | 1.000 | No difference |
|  | Diploma vs Illiterate | 52 | 10 | 0.627 | 0.001 | 0.079 | Trend (not significant) |
|  | Diploma vs Primary | 52 | 7 | 0.646 | 0.004 | 0.245 | Trend (not significant) |
| **EMPLOYMENT STATUS** | Unemployed vs Employed | 226 | 117 | 0.228 | 0.0004 | 0.057 | Trend toward significance |
|  | **Homemaker vs Employed** | **46** | **117** | **0.410** | **<0.001** | **0.004** | **Highly significant**** |
|  | Self-employed vs Employed | 20 | 117 | 0.350 | 0.010 | 1.000 | Not significant after correction |
|  | Student vs Employed | 28 | 117 | -0.043 | 0.707 | 1.000 | No difference |
|  | Retired vs Employed | 36 | 117 | 0.223 | 0.037 | 1.000 | Not significant after correction |
|  | Homemaker vs Student | 46 | 28 | 0.457 | 0.001 | 0.116 | Trend (not significant) |
|  | Self-employed vs Student | 20 | 28 | 0.389 | 0.020 | 1.000 | Not significant after correction |
|  | Student vs Unemployed | 28 | 226 | -0.273 | 0.017 | 1.000 | Not significant after correction |
|  | Retired vs Unemployed | 36 | 226 | 0.008 | 0.938 | 1.000 | No difference |
| **MARITAL STATUS** | **Divorced vs Married** | **42** | **307** | **0.267** | **0.004** | **0.022** | **Significant difference*** |
|  | **Divorced vs Single** | **42** | **109** | **0.398** | **0.0001** | **<0.001** | **Highly significant**** |
|  | Divorced vs Widowed | 42 | 32 | 0.060 | 0.659 | 0.659 | No difference |
|  | **Married vs Single** | **307** | **109** | **0.164** | **0.009** | **0.028** | **Significant difference*** |
|  | Married vs Widowed | 307 | 32 | -0.160 | 0.130 | 0.259 | No difference |
|  | **Single vs Widowed** | **109** | **32** | **-0.313** | **0.006** | **0.024** | **Significant difference*** |

***Statistical Notes:*** *All pairwise comparisons use Mann-Whitney U test****;*** *Holm sequential correction applied for multiple comparisons within each factor****;*** *Negative Cliff's δ indicates first group has lower HAQ-DI (less disability)****;*** *Positive Cliff's δ indicates first group has higher HAQ-DI (more disability)****. Significance Levels:*** ** P-value< 0.05,** P-value< 0.01, *** P-value< 0.001.* ***Abbreviations:*** *CI, confidence interval; HAQ-DI, Health Assessment Questionnaire Disability Index; R², coefficient of determination; δ, Cliff's delta effect size; Unadj, unadjusted P-value.*
